# Free-living physical activity and executive function: A multi-study analysis of age groups and times of day

**DOI:** 10.1016/j.ijchp.2023.100425

**Published:** 2023-12-06

**Authors:** Anne Eppinger-Ruiz de Zarate, Daniel Powell, Jan Kühnhausen, Julia L. Allan, Alexandra Johnstone, Daniel R. Crabtree, William Buosi, Claire L. Fyfe, David McMinn, Brett McCavour, Caterina Gawrilow, Gertraud Stadler

**Affiliations:** a Department of Psychology, University of Tuebingen, Tuebingen, Germany; b LEAD Graduate School & Research Network, University of Tübingen, Germany; c IDeA – Interdisciplinary Research Centre for Individual Development and Adaptive Education, Goethe University, Frankfurt, Germany; d Health Psychology, University of Aberdeen, Aberdeen, UK, AB25 2ZD; e Rowett Institute, University of Aberdeen, Aberdeen, UK, AB25 2ZD; f Department of Child and Adolescent Psychiatry, Psychosomatics and Psychotherapy, University Hospital Tuebingen, Germany; g Division of Psychology, University of Stirling, UK; h Gender in Medicine, Health & Human Sciences, Charite Universitaetsmedizin Berlin, Berlin, Germany; i German Centre for Mental Health (DZPG), Germany

**Keywords:** physical activity, accelerometer, cognitive function, ambulatory assessment

## Abstract

**Background:**

Executive Function (EF) is a potential mechanism linking physical activity (PA) and mental health. However, evidence regarding the association between free-living PA and EF is limited with mixed results. Across two studies, we examined associations between accelerometer-assessed moderate-to-vigorous PA (MVPA) and facets of EF in different age groups (Study 1) and at different times of day (Study 2).

**Method:**

In Study 1, we tested the association between MVPA and verbal fluency across seven days in 285 participants (children, adults, older adults). In Study 2, we tested between- and within-person associations between MVPA and working memory (afternoon, evening, next morning) across three 18-day bursts in 64 preadolescents.

**Results:**

Study 1 showed no association between MVPA and verbal fluency overall, but there was an interaction by age group: a positive association was evident in older adults only. In Study 2, we observed a positive between-person association between MVPA and subsequent afternoon and next morning working memory, but not within-person. In the evening, MVPA was not related to working memory.

**Conclusions:**

The association between free-living PA and EF differs between age groups and times of day. Future research should consider these factors when examining the association and its role for mental health.

## Physical activity and executive function: A multi-study analysis of age groups and times of day

Living a physically active life is of central importance for mental health across the lifespan. For example, physical activity (PA) interventions can prevent depression in the general population across different age groups (Hoare et al., 2021). While PA refers to any bodily movement increasing energy expenditure, moderate-to-vigorous PA (MVPA) is especially effective in the treatment of depression (Schuch et al., 2016).

One possible factor that mediates the link between PA and different mental health aspects (e.g., depressive symptoms) is executive function (EF; Dong et al., 2022): a set of higher order cognitive functions enabling individuals to direct and regulate their cognitions, emotions, and actions in a goal-oriented manner (Barkley, 2001). EF deficits can be observed for example in individuals with depression, and PA presents a promising nonpharmacological intervention to increase EF in this population (Ren et al., 2023). On the neurophysiological level, PA alters brain activity and connectivity, thereby improving EF (Erickson et al., 2015), which could indirectly explain the positive association between PA and mental health. Miyake et al. (2000) postulated the integrative model of EF that describes EF as consisting of one common underlying factor and three distinct components: cognitive flexibility, working memory (WM), and inhibition. EF undergoes a shift in development in preschool age, continuing into young adulthood (Huizinga et al., 2006). The age at which EF peaks and begins to decline depends on the specific EF component but all EF components show decreases in function with age, starting in midlife and progressing into older adulthood (Ferguson et al., 2021). In addition to age, individual characteristics (Kramer & Colcombe, 2018) and external influences also determine EF performance (Dirk & Schmiedek, 2017). EF fluctuates over time (McKinney et al., 2020) and is related to individuals’ engagement in effortful behaviours like PA.

## Relationship between physical activity and executive function

So far, most research has examined the relationship between PA and EF with interventional study designs (Donnelly et al., 2016). Reviews and meta-analyses report positive effects of PA interventions on EF across the lifespan (Alvarez-Bueno et al., 2017; Colcombe & Kramer, 2003; Donnelly et al., 2016; Verburgh et al., 2014). Ludyga et al. (2016) re-analyzed data from 40 interventional studies across the lifespan and found a small positive effect of PA on overall EF with strongest benefits in preadolescents and older adults. They explained this with higher sensitivity to PA in phases of developmental change.

While results from interventional research point towards a beneficial effect of PA on EF, these findings do not necessarily generalize to free-living PA and its implications for mental health. Yet, research examining the effect of free-living PA on EF is limited (Wickel, 2017). Across the lifespan, reviews suggest a positive link (Cox et al., 2016; Donnelly et al., 2016). Cross-sectional studies showed that objectively-measured total volume of PA was positively related to EF in children (van der Niet et al., 2015), and questionnaire-assessed free-living PA was positively related to EF in young adults (Kamijo & Takeda, 2010) and in older adults (Reas et al., 2019). However, very few studies used accelerometers as an objective and valid measure of free-living PA. Correlations between questionnaire- and accelerometer-derived PA are low-to-moderate (Prince et al., 2008), and differences between measurement methods possibly influence the observed relationship between PA and EF (Syväoja et al., 2014). Thus, more research examining the association between objectively measured free-living PA and EF is needed to better understand the role of an active lifestyle for EF (Donnelly et al., 2016).

## Present Research

We present two studies to examine different aspects of the relationship between free-living PA and key facets of EF as secondary analyses of two existing datasets: (1) a large cross-sectional study of verbal fluency (VF) and accelerometer-assessed PA across children, adults, and older adults; and (2) an intensive longitudinal study of spatial WM across different times of day (mornings, afternoons, evenings) and accelerometer-assessed PA in preadolescents.

## Study 1

Examining the role of free-living PA for EF across the lifespan is important, because evidence supports a causal positive effect of MVPA on cognitive functions (Cheval et al., 2023) and a longitudinal association between PA and EF, with strongest effects in lifelong active individuals (Reas et al., 2019). Further, findings from studies objectively measuring free-living PA and its relation to EF are mixed across different age groups. In preadolescent children, multiple studies reported no association between accelerometer-measured MVPA and cognitive flexibility (Syväoja et al., 2014; van der Niet et al., 2015), while in young adults, evidence suggests a positive association (Lin et al., 2018). In middle-aged and older adults, a study suggests that as little as ten minutes of MVPA per day were related to better cognitive flexibility (Spartano et al., 2019). The VF test implemented in study 1 is often considered to tap cognitive flexibility (Diamond, 2013) though VF has also been argued to involve a combination of general executive functions (e.g., Gustavson et al., 2019).

Since only a limited number of studies examined the relationship between objectively measured free-living PA and EF (Wickel, 2017), findings are inconsistent across age groups, and studies are conducted in different age groups rather than across multiple age groups, we wanted to examine this association across the lifespan. We hypothesised a positive association between free-living MVPA and VF as a measure of cognitive flexibility in children, adults, and older adults. We followed this up by exploring the interaction of this association by age, expecting to see a more positive association among adults.

## Method

### *Design & Participants*

Study 1 pools three datasets from larger projects (Full4Health: (Crabtree et al., 2020); Snapshot: (McMinn & Allan, 2014); StudentProject: (McCavour, 2018)) that all included the same EF task and objective measurement of free-living PA over seven days via accelerometers, resulting in a total sample of 285 participants. The description of each sample can be found in the Appendix.

We applied a minimum wear-time criterion of 4 days of data with at least 6 hours of wear-time per day, including at least one weekend day (Jerome et al., 2009). This led to the exclusion of 36 participants (Full4Health: 31, Snapshot: 2, StudentProject: 3), leaving a final pooled sample for analysis of 249 participants. Of these, 68 were children (under 18 yrs: *M* = 11.97 yrs, *SD* = 3.71 yrs), 144 adults (18-64 yrs: *M* = 34.33, *SD* = 13.35), and 36 older adults (65 yrs or older: *M* = 68.6, *SD* = 3.32). All three projects were approved by the University of Aberdeen Ethical Review Boards (CERB/2012/8/761; CERB/2018/2/1547) or the North of Scotland NHS Research Ethics Committee (12/NS/0007).

### *Measures*

**Executive Function.** We used the VF test of the Delis-Kaplan Executive Function System (D-KEFS; Delis et al., 2001), including the letter fluency task only. The letter fluency task is a 5-minute task requiring participants to name as many words as they can, in 60 seconds, starting with F, then A, then S. Participants are asked to avoid names, place names, numbers, repetitions, and the same word with different endings (e.g. slow, slowing). Raw scores are converted to scaled scores normed by age and gender, with higher scores reflecting better VF performance.

**Physical activity.** Free-living MVPA was assessed over seven consecutive days by Actigraph GT3X+ accelerometers (Actigraph, LLC, Fort Walton Beach, Florida) worn on the hip during waking hours. Bodily movements were measured on three axes with a frequency of 30 Hz and epoch length of one minute. For this analysis, Axis 1 counts were converted to minutes of MVPA using validated thresholds for adults (Freedson et al., 1998) and children (Freedson et al., 2005).

### *General Procedure*

After providing written informed (parental) consent, participants were asked to attend the University of Aberdeen to undertake a battery of cognitive tests, which included the D-KEFS letter fluency task. Children were generally tested in the school setting. Participants were shown how to wear the accelerometer and left the research site wearing it in the correct position. Formal data collection began the next day, and lasted for seven consecutive days. Upon completion, participants returned the devices to the researcher. In Full4Health and Snapshot, all participants had travel costs reimbursed; in StudentProject, participants received a £20 retail voucher plus travel costs.

### *Statistical Analysis*

To test the primary hypothesis, we used hierarchical linear regression to model VF performance as a function of minutes of MVPA, controlling for study number, age, gender, and accelerometer wear-time. MVPA data were heavily skewed, and therefore transformed using a log transformation (Log (MVPA + 1)). To test moderation by age, interactions of MVPA✗Age for each age group (children, adults, older adults) were examined using dummy variables. All analyses for Study 1 were conducted in SPSS using *ɑ* = .05 to denote statistical significance. All analyses of this two-study paper were pre-registered, and any deviations can be found in a brief document on Open Science Framework (OSF; https://osf.io/xyvk2/).

## Results

Descriptive statistics regarding time spent in MVPA, VF and wear-time can be found in the Appendix (Table A.1). In the linear regression, we observed no significant association between MVPA and VF across the age groups (*B* = 0.288, *SE* = 0.800, *p* = .719, 95% CI: -1.289, 1.865; See Table A.2, Appendix).

**Fig. 1:**
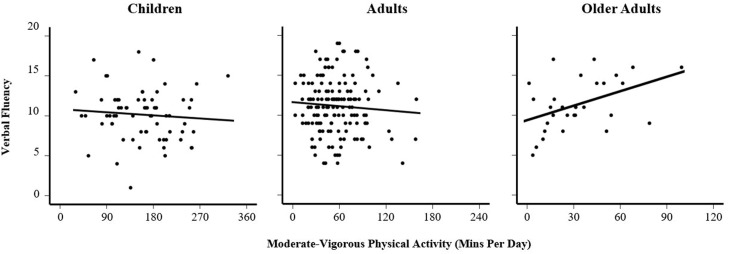
Association between Physical Activity and Verbal Fluency across different Age Groups

Examining the interaction by age group, it was evident that the association in older adults was significantly different to that in children (see Table 1; for full model see Table A.3 in Appendix) with a substantially more positive association in older adults (see Fig. 1).

**Table 1:** Linear Regression examining Verbal Fluency Performance as a Function of MVPA and its Interaction by Age Group

	Est.	SE	p
**Constant**	**11.289**	**0.927**	**< .001**
**Gender (Reference = Male)**	**-0.921**	**0.457**	**.045**
Adults (Reference = Children)	0.322	1.022	.748
Older Adults (Reference = Children)	2.180	1.254	.083
MVPA	-2.046	2.430	.314
MVPA * Adults	-0.879	2.284	.701
MVPA * Older Adults	**5.212**	**2.563**	**.043**

*Note*. MVPA is log-transformed and centered at the grand mean. Study covariates (study number, accelerometer wear-time) omitted, see Table A.3 for full results (Appendix).

## Discussion

Across different age groups, we found no association between time spent in MVPA and EF as measured by VF. This was unexpected, with previous research suggesting a positive association between objectively measured PA and EF across the lifespan (Cox et al., 2016; Donnelly et al., 2016). After including the interaction with age groups (children, adults, older adults), we only found a positive association between MVPA and VF in older adults. In children and adults, we found no association between MVPA and VF, supporting findings found elsewhere where no association was observed between MVPA and cognitive flexibility (e.g., Syväoja et al., 2014).

However, studies elsewhere looking at more specific age groups give a mixed picture. One study of free-living MVPA and various EF tasks found negative associations across all EF domains in 3-5 year olds (Willoughby et al., 2018); a study in 8-12 year olds found total volume of PA (including MVPA and light activity) was associated with better EF (van der Niet et al., 2015), and various papers suggest robust associations between MVPA and EF in older adulthood (Spartano et al., 2019; Zhu et al., 2017) including with the VF test (Daly, McMinn, & Allan, 2015). In a meta-analysis of moderate aerobic exercise effects on EF, small positive effects were observed for reaction time and accuracy measures (Ludyga et al., 2016) but moderation by age was evident in reaction time measures only: effects were strongest in older adults and preadolescents. Taken together, it appears likely any association is most-relevant in older adults, with some potential for specific effects on EF in children, though it appears not for VF; it is likely that VF captures elements of language proficiency over and above EF that may not yet be fully developed in children (Cohen et al., 1999).

To our knowledge, the sample in Study 1 is the largest dataset of its kind with objective and comparable measures of free-living PA and EF across different age groups. Study 1 has some methodological limitations: Firstly, our findings were only correlational. Secondly, we defined VF as a subdomain of EF in line with Diamond (2013) but this categorization is debated in the literature (e.g., Whiteside et al., 2016). When interpreting our findings, it needs to be considered that VF measures assess important core aspects of EF but also capture elements of language processing.

Our results from Study 1 highlight the importance of differentiating between age groups when examining the role of free-living PA for EF, which is supported by both interventional and observational research (Ludyga et al., 2016; Spartano et al., 2019; Syväoja et al., 2014). However, to better understand the association between PA and EF, research should not only consider age but also the time frame on which this relation occurs. Longitudinal evidence suggests positive effects of a lifelong active lifestyle (Reas et al., 2019). Thus, effects could accumulate over the life course, resulting in strongest associations in older adults. Objective measures of PA allow us to examine the association in a more natural setting and in more timely proximity, reflecting the increased variability of free-living PA (as compared to PA interventions).

## Study 2

In everyday life, PA and EF vary between and also within individuals over time (McKinney et al., 2020; Turrisi et al., 2021). Considering this variability is important when transferring results from interventional studies to a more natural setting. Findings regarding the relation between objectively-measured overall PA levels and WM performance are mixed: one study reported that preadolescents with low PA levels showed significantly worse WM accuracy (Zhu et al., 2022), while other evidence suggests no association between MVPA levels and preadolescents’ WM (Mücke et al., 2018; van der Niet et al., 2015). Insufficient control of confounding variables (e.g., general cognitive abilities; Hsieh et al., 2018), or of the variability in PA (Migueles et al., 2021) could explain null-findings and mixed results.

Previous research has shown that the effect of free-living PA differs depending on the investigated time interval: while one study reported beneficial effects of PA for same-evening affect (Haas et al., 2017), another study found no association with next-morning affect (Hachenberger et al., 2023). However, only a limited number of studies have considered the variability of PA when examining its role for EF. First evidence suggests that activity-rest-patterns – rather than overall PA levels – are related to WM performance in preadolescents (Migueles et al., 2021). Ambulatory assessment studies are particularly well-suited to capture such variability and different time scales, as they repeatedly measure individuals’ behaviour, permitting the separation of effects found between individuals (between-person) from those found within individuals over time (within-person). To our knowledge, only one ambulatory assessment study investigated MVPA and WM in preadolescents (Trevillion et al., 2022): over 14 days, 35 preadolescents wore an accelerometer and performed a WM task twice per school day. Higher levels of MVPA on the prior day were related to lower WM on the following day, but there was no association with MVPA in the two hours prior to WM assessment. These findings support a within-person relation between prior day MVPA and WM but not within the same day, highlighting the importance of examining the association between PA and EF on different time scales. However, Trevillion et al. examined a relatively small sample and collected data only twice a day for two weeks. Studies with larger sample sizes and more measurement occasions over a longer time period are necessary to better understand the associations between free-living PA and EF within and between individuals in everyday life and on different time scales.

Thus, in Study 2, we conducted an ambulatory assessment study to examine the relationship between objectively measured free-living PA and WM in preadolescents on three different time scales. During three 18-day data collection bursts, preadolescents completed a visual WM task in the morning, afternoon, and evening. This is a novel examination testing the role of PA at different times across the entire day over the course of one year, allowing us to draw conclusions about the variability of the association between PA and EF but also the stability of the examined effects. We used WM as a measure of EF in Study 2, because a recent meta-analysis (Alvarez-Bueno et al., 2017) suggested that WM might be especially sensitive to PA in youth. We expected that preadolescents’ MVPA would be positively related to their WM accuracy on the between- and within-person level at all times of day.

## Methods

### *Participants*

Preadolescents (10-13 yrs) were recruited at schools in Southwest Germany. Over the course of the study, 74 participants took part, but only 64 participants wearing an accelerometer were considered for this analysis (age: *M* = 10.75 yrs, *SD* = 0.49 yrs^1^; 26 male, 38 female). Due to dropouts between the measurement bursts, 15 participants were newly recruited before the second burst (participation in 1 burst *n* = 22, in 2 bursts *n* = 22, in 3 bursts *n* = 20).

### *Procedure*

Study 2 was part of the AttentionGO project implementing ambulatory assessment. It was funded by the German Research Foundation (project number GA 1277/9-1) and approved by the ethics committee of the German Society for Psychology (CG 102018_amd_112013). Recruitment in schools was approved by the Ministry of Culture, Youth, and Sport in Baden-Württemberg, Germany (file number 31-6499.20/1087). Data were collected in three separate 18-day measurement bursts approximately six months apart (Burst 1: November-December 2017; Burst 2: April-July 2018; Burst 3: November-December 2018).

At the start of each burst, children received smartphones and accelerometers. For each participant, one parent took part in a telephone interview (approx. 1 hr). During the 18 days, the smartphones rang three times a day for data collection and could not be turned on for another purpose. After ringing, children had 30 minutes to perform a spatial WM task. The time points all lay outside school hours: in the morning, afternoon, and evening. The exact times were individually agreed on with the parents at the beginning of each burst with possible differentiation between weekdays and weekends.

Participation was compensated with a 40-euro voucher for a chosen family activity (e.g., zoo visit) after each burst. Further, children received small presents and information about their PA.

## Material

### Daily Measures

***Executive Function.*** Participants completed a spatial WM task (Dirk & Schmiedek, 2016). They were presented with a matrix divided into 16 equal squares. Depending on the difficulty level, two (Load 2) or three (Load 3) virtual monsters were positioned on these squares and presented for 3000ms, followed by the presentation of the empty matrix for 250ms. After this, arrows appeared indicating in which direction the monsters moved. Each arrow was presented for 1500ms and followed by an inter stimulus interval (ISI) of 250ms. Two arrows were presented in Load 2 and, accordingly, three in Load 3. Finally, children indicated the monsters’ new positions (2000ms) and received feedback on accuracy after an ISI of 200ms. Children performed four trials of Load 2 followed by four trials of Load 3. In total, the task took approximately eight minutes.

As a measure for WM, accuracy was assessed in percentage. We only included Load 3 because of observed ceiling effects in Load 2. Accuracy was aggregated over the three answers within each trial of Load 3, and then averaged over all four trials of Load 3. Thus, WM-accuracy scores were computed for 54 measurement occasions per burst (3 per day for 18 days) and participant.

***Physical Activity.*** Participants wore an Actigraph GT3X+ on the hip of their non-dominant side during waking hours, with a frequency of 30 Hz and epoch length of 15 seconds. Axis 1 counts were converted to minutes of MVPA using validated thresholds (Evenson et al., 2008) and aggregated over one hour. Days with a wear-time of less than six hours were excluded (Jerome et al., 2009).

### Background Measures

***General cognitive abilities.*** We assessed general cognitive abilities through Raven's Standard Progressive Matrices (Horn, 2009) at the end of the first burst each child participated in and calculated the sum score.

***Demographic Data.*** Children's age and gender were collected in the parental interview.

### Data Analysis

All analyses for Study 2 were conducted with the statistical software R version 4.1.3 using *ɑ* = .05 to denote statistical significance. To test our hypotheses, we ran three multilevel linear regression models predicting WM performance in the (a) afternoon, (b) evening, and (c) next morning. Within the same day (a, b), PA was operationalised as hours spent in MVPA between 6am and the minute before the respective WM assessment. In the lagged analysis (c), we considered hours spent in MVPA between 6am and 11pm on the prior day. To distinguish between-person from within-person effects, we included two variables in the model: each child's mean hours spent in MVPA averaged across all bursts and centred on the grand mean to test the link of between-person differences in MVPA and EF; daily fluctuations in MVPA from 6am until the minute before the respective WM assessment at each study day centred around each child's person mean to test the within-person link between fluctuations in MVPA and EF. To account for possible training effects in the WM task, we included a linear time trend of study day (range 0-1) and dummy-coded variables representing the burst. We controlled for weekend, gender and wear-time (in hrs), age at Burst 1 and general cognitive abilities (centred on grand mean). We estimated random effects for the intercept, the within-person effect of MVPA, study day, the continuous autocorrelation of Level 1 residuals, and correlations between random effects. The equations describing the full models can be found in the Appendix.

## Results

**Descriptive Results.** Descriptive statistics regarding wear-time, time spent in MVPA and WM performance can be found in the Appendix (Tables A.4, A.5). MVPA varied across bursts (in mins; Burst 1: *M* = 48.16*, SD* = 29.01*;* Burst 2: *M* = 60.97*, SD* = 46.96*;* Burst 3: *M* = 47.41*, SD* = 25.39) with highest MVPA levels in Burst 2, and it differed between gender (in mins; girls: *M* = 46.38, *SD* = 31.06; boys: *M* = 60.87, *SD* = 40.33) with higher MVPA levels in boys. WM performance differed between bursts (in percentage; Burst 1: *M* = 56.32*, SD* = 29.29*;* Burst 2: *M* = 60.72*, SD* = 29.48*;* Burst 3: *M* = 65.53*, SD* = 26.99). Girls (*M* = 62.68, *SD* = 28.70) had higher scores, on average, than boys (*M* = 56.12, *SD* = 29.09) across all times of day and bursts.

**Fig. 2:**
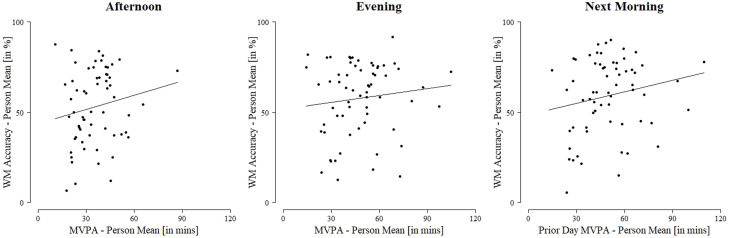
Between-Person Association between Physical Activity per Day (Person Mean) and Working Memory (Person Mean) across different Times of Day

**Multilevel Analyses.** The results of the multilevel models concerning the hypotheses as well as significant control variables are reported in Table 2 (for full model see Table A.6 in Appendix), and between-person associations are represented in Fig. 2.

**Table 2:** Physical Activity (MVPA in hrs) predicting Working Memory (in %) at different Times of Day while controlling for Study Day, Weekend, Wear-Time, Gender, Age, and General Cognitive Abilities

			Afternoon		Evening		Next Morning	
Fixed Effects			Est.	SE	Est.	SE	Est.	SE
Burst 1 (Reference)								
	Starting point	γ_00_	**42.26**	3.85***	**48.74**	3.76***	**49.47**	3.95***
	MVPA, between-person effect	γ_01_	**24.90**	11.52*	12.39	7.77	**17.16**	8.08*
	MVPA, within-person effect	γ_10_	0.01	3.84	-5.20	2.72	-0.21	2.89
Difference in Burst 2 vs. 1								
	Starting point	γ_30_	**5.59**	2.19*	**4.51**	2.15*	3.03	2.25
	MVPA, between-person effect	γ_31_	-2.35	8.35	-2.46	6.46	-1.51	6.72
	MVPA, within-person effect	γ_50_	-3.22	4.33	4.42	3.20	-0.78	3.43
Difference in Burst 3 vs. 1								
	Starting point	γ_40_	**8.92**	2.41***	**9.98**	2.34***	**8.69**	2.39***
	MVPA, between-person effect	γ_41_	-8.06	9.10	-6.79	6.75	-6.63	6.89
	MVPA, within-person effect	γ_60_	-2.41	6.35	6.08	4.59	-1.23	4.90
Adjustment variables								
	Weekend	γ_80_	0.48	2.23	**-3.73**	1.90	-0.78	2.03
	Gender (female)	γ_03_	**13.76**	4.93**	**10.75**	4.60	**13.65**	4.89**
	Age	γ_02_	-2.48	2.19	-3.24	2.13	**-4.84**	2.24*
	General cognitive abilities	γ_04_	**6.95**	2.24**	**5.51**	2.15*	**6.28**	2.27**
N_observations_		1060		1262		992		
N_participants_		63		64		63		

*Note*. *** *p* < .001, ** *p* < .01, * *p* < .05. Study covariates (study day, accelerometer wear-time) omitted, see Table A.6 for full results (Appendix).

***Afternoon Analysis.*** We found a significant positive between-person relationship between MVPA and afternoon WM performance (*γ_01_* = 24.90, *p* = .035). This did not change across the bursts, implying a stable positive between-person association. However, we did not find this association on the within-person level (*γ_10_* = 0.01, *p* = .999). We observed a significant increase in WM between bursts (Burst 2: *γ_30_* = 5.59, *p* = .011; Burst 3: *γ_40_* = 8.92, *p* < .001).

***Evening Analysis.*** We found no between-person association between MVPA and evening WM performance (*γ_01_* = 12.39, *p* = .116), and, after including autocorrelation, the negative within-person association failed to reach significance (*γ_10_* = -5.20, *p* = .057). Again, we found a significant increase in evening WM between bursts (Burst 2: *γ_30_* = 4.51, *p* = .036; Burst 3: *γ_40_* = 9.98, *p* < .001).

***Morning Analysis.*** We observed a significant positive between-person relationship between prior day MVPA and morning WM performance (*γ_01_* = 17.16, *p* = .038) that did not significantly change over the bursts. On the within-person level, we did not find an association (*γ_10_* = -0.21, *p* = .942). Morning WM significantly increased from Burst 1 to Burst 3 (*γ_40_* = 8.69, *p* < .001).

## Discussion

In Study 2, we found stable positive between-person associations between MVPA and WM in the afternoon and morning, in line with previous research (e.g., Hsieh et al., 2018). The design of Study 2 expands previous findings through information about the stability of the observed effects, as the relation between MVPA and EF was stable across a longer period in preadolescence and different seasons (Burst 1, 3: winter; Burst 2: summer). In the evening, we did not find a between-person association between MVPA and WM performance, contradicting our hypothesis but in line with some previous research (e.g., van der Niet et al., 2015). In children, EF performance is typically not measured in the evening. Thus, findings from other studies (e.g., Trevillion et al., 2022) might not be applicable to the evening WM assessment in our study. Before the evening assessment, daily structures and routines might have strongly varied between individuals, influencing the relationship between MVPA and WM. Hence, future studies examining the role of PA for evening WM should assess such contextual variables.

On the within-person level, we found no associations between MVPA and WM at any time of day. This contradicted our hypotheses but was somewhat in line with findings from the ambulatory assessment study by Trevillion et al. (2022) which found that MVPA in the two hours prior to WM assessment was not related to WM. Regarding prior day MVPA, they reported a negative association with WM while we found no association. Inconsistencies between the two studies might be explained through methodological aspects (e.g., different WM tasks). Contradicting our hypothesis, we observed a trend towards a negative association in the evening. Increased fatigue in the evening after more PA on the respective day (Haas et al., 2017) might negatively influence EF performance and explain this result pattern.

As in Study 1, our findings were only correlational. Still, the design of Study 2 offers considerable benefits yielding novel insights: As an ambulatory assessment study, Study 2 ensures a high ecological validity and reflects variability occurring in everyday life. This enables us to better understand the *real-life* and *real-time* association between individuals’ PA and their EF. Further, the examination of preadolescents’ WM in the evening granted us novel insights into the association between PA and EF beyond previous examinations (e.g., Trevillion et al., 2022). The association between PA and EF in the evening varied from the morning and afternoon associations, and more research is necessary to better understand why the association differs between times of day.

## General Discussion

The two studies presented in this paper offer an overarching examination of the relationship between objectively measured free-living PA and EF across the life span. The aim was to better understand the relationship between PA and EF in everyday life, by examining the relation in different age groups (Study 1) and on different time scales (Study 2). Study 1 revealed differences in the relationship between age groups: in line with previous findings (Ludyga et al., 2016), we observed strongest associations in older adults (positive association). In Study 2, we focused on preadolescents as an age group with limited previous research findings, and found differences between time of assessment: while we found no association in the evening, MVPA was positively related to subsequent WM in the afternoon and next morning between individuals. A key finding of Study 2 was that these associations were all stable across three measurement bursts spanning one year. The observed differences in the relationship between PA and EF in children may be explained with WM (Study 2) being more sensitive to PA in children than VF (Study 1; Alvarez-Bueno et al., 2017).

It is a strength of our multi-study paper that we employed objective measures of PA (accelerometer) and EF (objective tests of VF and WM), expanding previous research that often relied on interventional study designs (Wickel, 2017) or questionnaire-assessed PA (Donnelly et al., 2016). For Study 1 we examined VF, as the VF Test (Delis et al., 2001) was previously scaled across the lifespan. However, VF possibly captures language proficiency yet not fully developed in children (Cohen et al., 1999), thereby underestimating the association between PA and EF. Thus, in Study 2, we examined WM as a specifically sensitive measure in children (Alvarez-Bueno et al., 2017), although somewhat limitting the comparability between the two studies.

Despite the advantages of objectively measured PA, investigations of qualitative characteristics of PA and their relation to EF could help explain inconsistent research findings (van der Niet et al., 2015). Thus, future research could combine objective measures of PA with questionnaires. Further, our findings from Study 2 highlight the importance of employing more ambulatory assessment studies in the future to better understand the relation between PA and EF between and within individuals but also on different time scales. It could be especially interesting for future studies to examine the role of PA for evening EF. Ambulatory assessment studies should also be implemented in different age groups - especially in those with fast developmental changes (e.g., older adults). Future work should further implement comparable EF measures across the lifespan (Study 1) to increase comparability between different age groups.

Our findings implicate that free-living PA is of high relevance for EF, which, again, is relevant for individuals to engage in health behaviours (Dong et al., 2022). EF are closely linked to individuals’ mental health (e.g., depressive symptoms), and PA could be a promising solution to increase EF and therefore improve diverse mental health aspects (Ren et al., 2023). Investigating the importance of a physically active lifestyle is becoming increasingly important, and our paper helps generate hypotheses for future studies regarding differences between age groups as well as time of day. More research is needed to fully understand the relation between PA and EF in everyday life, and the role they can play for mental health.

## Funding

This paper was supported by the Scottish Government, Rural and Environmental Science & Analytical Services (RESAS) division. The Full4Health project was funded by the European Union's Seventh Framework Programme FP7-KBBE-2010-4 under grant agreement No: 266408. Authors from the University of Aberdeen, Rowett Institute gratefully acknowledge financial support from the Scottish Government as part of the RESAS Strategic Research Programme at the Rowett Institute. The AttentionGO project was supported by the Deutsche Forschungsgemeinschaft (GA1277/ 9-1). Gertraud Stadler gratefully acknowledges financial support from the Chancengleichheits-Programm. We acknowledge support from the Open Access Publication Fund of the University of Tübingen.

## CRediT authorship contribution statement

**Anne Eppinger-Ruiz de Zarate:** Data curation, Formal analysis, Investigation, Methodology, Visualization, Writing – original draft. **Daniel Powell:** Conceptualization, Data curation, Formal analysis, Methodology, Visualization, Writing – original draft. **Jan Kühnhausen:** Conceptualization, Data curation, Methodology, Writing – review & editing, Formal analysis. **Julia L. Allan:** Conceptualization, Writing – review & editing, Funding acquisition, Methodology. **Alexandra Johnstone:** Writing – review & editing, Funding acquisition. **Daniel R. Crabtree:** Writing – review & editing, Investigation. **William Buosi:** Writing – review & editing, Investigation. **Claire L. Fyfe:** Writing – review & editing, Investigation. **David McMinn:** Data curation, Writing – review & editing, Investigation, Methodology. **Brett McCavour:** Writing – review & editing, Investigation. **Caterina Gawrilow:** Conceptualization, Funding acquisition, Methodology, Supervision, Writing – review & editing, Project administration. **Gertraud Stadler:** Conceptualization, Methodology, Writing – review & editing, Supervision, Project administration, Formal analysis.

## Declaration of Competing Interest

The authors declare that they have no known competing financial interests or personal relationships that could have appeared to influence the work reported in this paper.

## Appendix Supplementary materials

Image, application 1

## Footnotes

1 For one subject, no age data was available. This child's age was estimated for the models.
