# Supplementary material for: Free-living physical activity and executive function: A multi-study analysis of age groups and times of day
Source: Int J Clin Health Psychol. 2023 Dec 6;24(1):100425. doi: 10.1016/j.ijchp.2023.100425 (PMC10714236; doi:10.1016/j.ijchp.2023.100425)
Supplement: Supplementary file 1 [file mmc1.docx]

**Appendix A: Supplementary Material**

Eppinger Ruiz de Zarate, A., Powell, D., Kühnhausen, J., Allan, J. L., Johnstone, A., Crabtree, D. R., Buosi, W., Fyfe, C. L., McMinn, D., McCavour, B., Gawrilow, C., & Stadler, G. (2023). Free-living physical activity and executive function: A multi-study analysis of age groups and times of day. *IJCHP, 24*(1), 100425.

**Study 1**

**Sample Descriptives Study 1**

### ***Participants***

***Sample 1.*** The Full4Health study (Amin & Mercer, 2016; Crabtree et al., 2020) was an EU-wide collaborative project primarily interested in food-gut-brain mechanisms, with the site collecting PA and CF data between 2013-2015. Participants were 97 children (51 females, 44 males, 2 not reported; *M_Age_* = 12.58 yrs, *SD_Age_* = 3.39 yrs, Range 7 – 17 yrs) and 86 adults (57 females, 29 males; *M_Age_* = 46.28 yrs, *SD_Age_* = 20.08 yrs, Range = 18 – 76 yrs). Participants were included if they were normal weight or overweight/obese, did not smoke, and had no history of major depressive disorder, cardiovascular or cerebrovascular disease, or chronic pulmonary disease. Ethical approval was granted by the North of Scotland Research Ethics Service (12/NS/0007).

***Sample 2.*** The Snapshot study (Allan et al., 2019; McMinn & Allan, 2014; Powell et al., 2017) dataset includes 68 adults (53 females, 15 males; *M_Age_* = 39.41 yrs, *SD_Age_* = 15.54 yrs) recruited in 2012-2014. All participants were recruited from the local community, fluent in the English language, and had no visual or motor impairments. Ethical approval was granted by the North of Scotland Ethics Review Board (CERB/2012/8/761).

***Sample 3.*** The final sample comes from an unpublished student (McCavour, 2018) project with 34 adults (21 females, 13 males; *M_Age_* = 28.79 yrs, *SD_Age_* = 11.02 yrs) collected in 2018. All participants were recruited from the local community, fluent in the English language, and had intended to perform MVPA in the week in question. Ethical approval was granted by the North of Scotland Ethics Review Board (CERB/2018/2/1547).

***Pooled sample.*** Across the three samples, 285 participants undertook the same CF task (details below) and same PA monitoring using accelerometers (details below). We applied a minimum wear-time criterion of at least 4 days of data with at least 6 hours of wear-time, including at least one weekend (Jerome et al., 2013). This led to the exclusion of 36 participants (Full4Health: 31, Snapshot: 2, StudentProject: 3) leaving a final pooled sample for analysis of 249 participants.

**Results Study 1**

***Descriptive Results***

**Table A.1.** Study 1 Descriptive Statistics

|  | *M* | *SD* | Median | Range |
| --- | --- | --- | --- | --- |
| MVPA (mins) | 81.69 | 63.19 | 59.86 | 1.14 – 324.20 |
| Verbal Fluency | 10.91 | 3.38 | 11.00 | 1.00 – 19.00 |
| Accelerometer Wear-Time (hrs) | 12.29 | 1.82 | 12.27 | 7.30 – 23.00 |

***Linear Regression models***

**Table A.2.** Linear regression examining CF performance as a function of MVPA

|  | *B* | *SE* | *p* |
| --- | --- | --- | --- |
| **Constant** | **11.320** | **0.388** | **< .001** |
| **Age** | **0.039** | **0.015** | **.010** |
| **Gender (Ref = Male)** | **-0.952** | **0.454** | **.037** |
| MVPA | 0.288 | 0.800 | .719 |
| Study 2 (Ref = Study 1) | 0.034 | 0.510 | .947 |
| Study 3 (Ref = Study 2) | 1.028 | 0.663 | .122 |
| Accelerometer Wear-Time | -0.154 | 0.126 | .223 |

*Note.* MVPA is log-transformed and centred at the grand mean. Age and wear-time are centred at the grand mean.

**Table A.3.** Linear regression examining CF performance as a function of MVPA and its interaction by age group.

|  | *B* | *SE* | *p* |
| --- | --- | --- | --- |
| **Constant** | **11.289** | **0.927** | **< .001** |
| Adults (Ref = Children) | 0.322 | 1.022 | .748 |
| Older Adults (Ref = Children) | 2.180 | 1.254 | .083 |
| **Gender (Ref = Male)** | **-0.921** | **0.457** | **.045** |
| MVPA | -2.046 | 2.430 | .314 |
| MVPA * Adults | 0.879 | 2.284 | .701 |
| **MVPA * Older Adults** | **5.212** | **2.563** | **.043** |
| Study 2 (Ref = Study 1) | -0.086 | 0.603 | .887 |
| Study 3 (Ref = Study 1) | 0.638 | 0.778 | .413 |
| Accelerometer Wear-Time | -0.168 | 0.129 | .194 |

*Note.* MVPA is log-transformed and centred at the grand mean. Wear-time is centred at the grand mean.

**Study 2**

**Descriptive Results Study 2**

Wear-time (*M* = 12.48 hrs, *SD* = 2.61 hrs) did not significantly differ between bursts (*F*(2, 1529) = 0.337, *p* = .714). The analysis of missing data revealed that across bursts wear-time was significantly correlated with gender (*t*(1309.9) = -3.857, *p* < .001; *M_male_* = 12.16 hrs, *SD* = 2.62 hrs; *M_female_* = 12.69 hrs, *SD* = 2.58 hrs), age (*r* = .06, *p* = .021) and weekend (*t*(565.49) = 12.753, *p* < .001; weekend: *M* = 10.98 hrs, *SD* = 2.50 hrs; weekday: *M* = 12.92 hrs, *SD* = 2.47 hrs). Thus, gender, age, and weekend were included as control variables in the models.

Descriptive statistics regarding time spent in MVPA are depicted in Table A.4. MVPA significantly varied across bursts (*F*(2, 1528) = 26.77, *p* < .001) with highest MVPA levels in burst 2, and it differed between gender (*F*(1, 1528) = 65.03, *p* < .001) with higher MVPA levels in boys.

**Table A.4.** Average time spent in MVPA (in mins) across bursts and gender.

|  |  | Burst 1 | | Burst 2 | | Burst 3 | |
| --- | --- | --- | --- | --- | --- | --- | --- |
|  | | *M* | *SD* | *M* | *SD* | *M* | *SD* |
| Time spent in MVPA | | 48.16 | 29.01 | 30.97 | 46.96 | 47.41 | 25.39 |
|  | Female | 42.88 | 24.62 | 54.17 | 40.84 | 41.18 | 20.99 |
|  | Male | 55.40 | 32.81 | 72.27 | 53.89 | 56.43 | 28.41 |

Descriptive results regarding WM can be found in Table A.5. Across times of day, WM performance differed significantly between bursts (afternoon: *F*(2, 1061) = 7.04, *p* < .001; evening: *F*(2, 1264) = 9.01, *p* < .001; morning: *F*(2, 1172) = 13.84, *p* < .001) and gender (afternoon: *F*(1, 1061) = 10.38, *p* = .001; evening: *F*(1, 1264) = 21.28, *p* < .001; morning: *F*(1, 1172) = 14.10, *p* < .001). Across all times of day and bursts, girls scored significantly higher than boys and WM performance was positively related to general cognitive abilities (*ρ_afternoon_* = .40, *p* = .001; *ρ_evening_* = .31, *p* = .012; *ρ_morning_* = .41, *p* < .001). Thus, general cognitive abilities were also included as a control variable in the models.

**Table A.5.** Average WM performance (in %) across bursts, gender, and time of day.

|  |  | Afternoon | | Evening | | Morning | |
| --- | --- | --- | --- | --- | --- | --- | --- |
|  | | *M* | *M* | *M* | *M* | *M* | *SD* |
| Burst 1 | | 57.41 | 53.25 | 57.92 | 57.41 | 57.41 | 29.51 |
|  | Female | 58.12 | 61.60 | 61.25 | 61.25 | 61.60 | 28.41 |
|  | Male | 46.63 | 52.93 | 52.05 | 52.05 | 52.93 | 30.29 |
| Burst 2 | | 62.12 | 57.91 | 61.77 | 62.12 | 62.12 | 28.72 |
|  | Female | 56.88 | 63.66 | 62.37 | 62.37 | 63.66 | 29.24 |
|  | Male | 59.45 | 58.95 | 61.77 | 61.77 | 58.95 | 27.78 |
| Burst 3 | | 68.05 | 61.47 | 66.50 | 68.05 | 68.05 | 26.65 |
|  | Female | 64.35 | 70.14 | 72.03 | 72.03 | 70.14 | 25.24 |
|  | Male | 57.89 | 61.48 | 63.11 | 63.11 | 61.48 | 27.80 |

**Multilevel Analyses in Study 2**

To examine the relation between MVPA and WM performance on different time scales, we measured WM in the afternoon, evening, and the next morning. Further, we considered MVPA in different time windows for the analysis: (a) for the afternoon analysis, we considered MVPA between 6am and the minute before the afternoon WM assessment (typically between 1:30 and 5:30 pm); (b) for the evening analysis, we included MVPA between 6am and the minute before the evening WM assessment (school days: typically between 7:30 and 9:30 pm; weekends: typically between 7:30 and 11:00 pm); and (c) for the lagged analysis predicting WM the next morning (school days: typically between 6:00 and 7:45 am; weekends: typically between 7:00 and 10:30), we considered prior day MVPA between 6am and 11pm. To investigate these time scales separately, we conducted three individual multilevel models.

The multilevel models examining the relation between MVPA and WM within the same day (afternoon, evening) included the following variables: each child *i*’s person mean hours spent in MVPA between 6am and the respective WM assessment (in the afternoon or evening) averaged across all bursts and centred on the grand mean (mMVPA*_i_*) to test the link of between-person differences in MVPA and EF on Level 2. Daily fluctuations in MVPA from 6am until right before the respective WM assessment at study day *t* around each child’s person mean (rMVPA*_ti_*) to test the within-person link between fluctuations in MVPA and EF on Level 1. We included a linear time trend of study day (time*_ti_*; range 0-1) and dummy-coded variables representing the burst (B2*_ti_*, B3*_ti_*). We controlled for weekend, gender and wear-time (in hrs), age at Burst 1 and general cognitive abilities (centred on grand mean). We estimated random effects for the intercept (υ_o_*_i_*), the within-person effect of MVPA (υ_1_*_i_*), study day (υ_2_*_i_*), the continuous autocorrelation of Level 1 residuals, and correlations between random effects. Equation (A.1) describes the full same-day models:

| (A.1) | WM_ti_ = | γ_00_ + γ_01_ ∙ mMVPA*_i_* + (γ_30_ + γ_31_ ∙ mMVPA*_i_*) ∙ B2*_ti_* + (γ_40_ + γ_41_ ∙ mMVPA*_i_*) ∙ B3*_ti_* + (γ_10_ + υ_1_*_i_*) ∙ rMVPA*_ti_* + γ_50_ ∙ B2*_ti_* × rMVPA*_ti_* +γ_60_ ∙ B3*_ti_* × rMVPA*_ti_*  + (γ_20_ + υ_2_*_i_*) ∙ time*_ti_* + γ_70_ ∙ wear-time*_ti_* + γ_80_ ∙ weekend*_ti_* + γ_02_ ∙ age*_i_* + γ_03_ ∙ gender*_i_* + γ_04_ ∙ cognitive abilities*_i_* + υ_o_*_i_* + e*_ti_* |
| --- | --- | --- |

## In the lagged analysis examining the relation between prior day MVPA and WM performance in the morning of study day *t*, we included the following variables: each child *i*’s person mean hours spent in MVPA between 6am and 11pm averaged across all bursts and centred on the grand mean (mMVPA*_i_*) to test the link of between-person differences in MVPA and EF on Level 2. Daily fluctuations in MVPA between 6am and 11pm of the prior study day *t-1* around each child’s person mean (rMVPA*_t-1i_*) to test the link between within-person fluctuations in MVPA on one day and EF the next morning on Level 1. The control variables (study day, burst, weekend, gender, wear-time, age at Burst 1, general cognitive abilities) and random effects (intercept, within-person effect of MVPA, study day, continuous autocorrelation of Level 1 residuals) were the same as in the same-day multilevel models (afternoon, evening). Equation (A.2) describes the full model for the lagged analysis:

| (A.2) | WM_ti_ = | γ_00_ + γ_01_ ∙ mMVPA*_i_* + (γ_30_ + γ_31_ ∙ mMVPA*_i_*) ∙ B2*_ti_* + (γ_40_ + γ_41_ ∙ mMVPA*_i_*) ∙ B3*_ti_* + (γ_10_ + υ_1_*_i_*) ∙ rMVPA*_t-1i_* + γ_50_ ∙ B2*_ti_* × rMVPA*_t-1i_* +γ_60_ ∙ B3*_ti_* × rMVPA*_t-1i_*  + (γ_20_ + υ_2_*_i_*) ∙ time*_ti_* + γ_70_ ∙ wear-time*_ti_* + γ_80_ ∙ weekend*_ti_* + γ_02_ ∙ age*_i_* + γ_03_ ∙ gender*_i_* + γ_04_ ∙ cognitive abilities*_i_* + υ_o_*_i_* + e*_ti_* |
| --- | --- | --- |

##

**Table A.6.** *Physical Activity (MVPA in hrs) predicting working memory (in percentage) at different times of day while controlling for study day, weekend, wear time, gender, age, and general cognitive abilities.*

|  |  |  | Afternoon | | | Evening | | | Next Morning | | |
| --- | --- | --- | --- | --- | --- | --- | --- | --- | --- | --- | --- |
| Fixed Effects | |  | *Est.* |  | *SE* | Est. |  | *SE* | Est. |  | *SE* |
| Burst 1 (Reference period) | |  |  |  |  |  |  |  |  |  |  |
|  | Starting point | γ_00_ | **42.26** | *** | 3.85 | **48.74** | ******* | 3.76 | **49.47** | *** | 3.95 |
|  | MVPA, between-person effect | γ_01_ | **24.90** | * | 11.52 | 12.39 |  | 7.77 | **17.16** | * | 8.08 |
|  | MVPA, within-person effect | γ_10_ | 0.01 |  | 3.84 | -5.20 |  | 2.72 | -0.21 |  | 2.89 |
| Difference in Burst 2 vs. 1 | |  |  |  |  |  |  |  |  |  |  |
|  | Starting point | γ_30_ | **5.59** | * | 2.19 | **4.51** | ***** | 2.15 | 3.03 |  | 2.25 |
|  | MVPA, between-person effect | γ_31_ | -2.35 |  | 8.35 | -2.46 |  | 6.46 | -1.51 |  | 6.72 |
|  | MVPA, within-person effect | γ_50_ | -3.22 |  | 4.33 | 4.42 |  | 3.20 | -0.78 |  | 3.43 |
| Difference in Burst 3 vs. 1 | |  |  |  |  |  |  |  |  |  |  |
|  | Starting point | γ_40_ | **8.92** | *** | 2.41 | **9.98** | ******* | 2.34 | **8.69** | *** | 2.39 |
|  | MVPA, between-person effect | γ_41_ | -8.06 |  | 9.10 | -6.79 |  | 6.75 | -6.63 |  | 6.89 |
|  | MVPA, within-person effect | γ_60_ | -2.41 |  | 6.35 | 6.08 |  | 4.59 | -1.23 |  | 4.90 |
| Adjustment variables | |  |  |  |  |  |  |  |  |  |  |
|  | Study Day | γ_20_ | -0.88 |  | 3.03 | 1.67 |  | 2.96 | -2.00 |  | 3.03 |
|  | Weekend | γ_80_ | 0.48 |  | 2.23 | **-3.73** | * | 1.90 | -0.78 |  | 2.03 |
|  | Wear-Time | γ_70_ | 0.00 |  | 0.46 | 0.35 |  | 0.37 | -0.02 |  | 0.36 |
|  | Gender (female) | γ_03_ | **13.76** | ** | 4.93 | **10.75** | * | 4.60 | **13.65** | ** | 4.89 |
|  | Age | γ_02_ | -2.48 |  | 2.19 | -3.24 |  | 2.13 | **-4.84** | * | 2.24 |
|  | General cognitive abilities | γ_04_ | **6.95** | ** | 2.24 | **5.51** | * | 2.15 | **6.28** | ** | 2.27 |
| Random effects | |  |  |  |  |  |  |  |  |  |  |
| *Level 2 (between-person)* | |  |  |  |  |  |  |  |  |  |  |
|  | Intercept | *SD*(υ_o_*_i_*) | **14.34** | *** |  | **15.56** | ******* |  | **15.99** | *** |  |
|  | MVPA, within-person effect | *SD*(υ_1_*_i_*) | 3.74 |  |  | 1.30 |  |  | 1.97 |  |  |
|  | Study Day | *SD*(υ_2_*_i_*) | 10.10 |  |  | 10.97 |  |  | 10.89 |  |  |
|  | Intercept with MVPA | *r*(υ_0_*_i,_* υ_1_*_i_*) | 0.26 |  |  | -0.27 |  |  | -0.26 |  |  |
|  | Intercept with Study Day | r(υ_0_*_i,_* υ_2_*_i_*) | 0.19 |  |  | -0.28 |  |  | -0.23 |  |  |
|  | MVPA with Study Day | *r*(υ_1_*_i,_* υ_2_*_i_*) | 0.94 |  |  | 0.94 |  |  | 0.44 |  |  |
| *Level 1 (within-person)* | |  |  |  |  |  |  |  |  |  |  |
|  | Residual | *SD*(ε*_ti_*) | 23.56 |  |  | 23.55 |  |  | 21.78 |  |  |
|  | Autocorrelation | *ρ* | 0.06 |  |  | **0.11** | *** |  | **0.08** | ***** |  |
| *N_observations_* | |  | 1060 |  |  | 1262 |  |  | 992 |  |  |
| *N_participants_* | |  | 63 |  |  | 64 |  |  | 63 |  |  |

*Note*. *** *p* < .001, ** *p* < .01, * *p* < .05.

***Afternoon Analysis***

Afternoon WM performance was associated with gender, with girls performing better than boys (*γ_03_* = 13.67 (*SE* = 4.93), *p* = .008), and positively associated with general cognitive abilities (*γ_04_* = 6.95 (*SE* = 2.24), *p* = .003). We observed a significant random effect for intercept, indicating a considerable variance in children’s initial WM (*SD*(υ_o_*_i_*) = 14.34, *p* < .001).

***Evening Analysis***

Evening WM performance was significantly lower on the weekends (*γ_80_* = -3,73 (*SE* = 1.90), *p* = .050), and significantly higher in girls (*γ_03_* = 10.75 (*SE* = 4.60), *p* = .023). It was further positively related to general cognitive abilities (*γ_04_* = 5.51 (*SE* = 2.15), *p* = .013). We observed a significant random effect for intercept (*SD*(υ_o_*_i_*) = 15.56, *p* < .001) and a significant autocorrelation (*ρ* = 0.11, *p* < .001).

***Morning Analysis***

Morning WM performance was related to gender (*γ_03_* = 13.65 (*SE* = 4.89), *p* = .007), with girls showing higher WM scores than boys, and general cognitive abilities (*γ_04_* = 6.28 (*SE*= 2.27), *p* = .008). We also observed a significant negative effect of age (*γ_02_* = -4.84 (*SE* = 2.24), *p* = .035). Again, we found a significant random effect for intercept (*SD*(υ_o_*_i_*) = 15.99, *p* < .001) and a significant autocorrelation (*ρ* = 0.08, *p* = .041).

**References**

Allan, J., Mcminn, D., & Powell, D. (2019). Tracking snacking in real time: Time to look at individualised patterns of behaviour. *Nutr Health*, *25*(3), 179–184. https://doi.org/10.1177/0260106019866099

Amin, T., & Mercer, J. G. (2016). Full4Health: Understanding food-gut-brain mechanisms across the lifespan in the regulation of hunger and satiety for health. *Nutrition Bulletin*, *41*(1), 87–91. https://doi.org/10.1111/nbu.12193

Crabtree, D. R., Buosi, W., Fyfe, C. L., Horgan, G. W., Manios, Y., Androutsos, O., Giannopoulou, A., Finlayson, G., Beaulieu, K., Meek, C. L., Holst, J. J., Van Norren, K., Mercer, J. G., & Johnstone, A. M. (2020). Appetite control across the lifecourse : The Acute impact of breakfast drink quantity and protein content. The Full4Health Project. *Nutrients*, *12*, 3710. https://doi.org/10.3390/nu12123710

McCavour, B. (2018). *Which facets of executive function relate most strongly to physical activity behaviour?* University of Aberdeen.

McMinn, D., & Allan, J. L. (2014). The SNAPSHOT study protocol: SNAcking, Physical activity, Self-regulation, and Heart rate Over Time. *BMC Public Health*, *14*(1), 1–11. https://doi.org/10.1186/1471-2458-14-1006

Powell, D., McCinn, D., & Allan, J. L. (2017). Does real time variability in inhibitory control drive snacking behavior? An intensive longitudinal study. *Health Psychology*, *36*(4), 356–364. https://doi.org/10.1037/hea0000471
